# Enzymatic Fructosylation of Phenolic Compounds: A New Alternative for the Development of Antidiabetic Drugs

**DOI:** 10.3390/molecules29133072

**Published:** 2024-06-27

**Authors:** Karla Damian-Medina, Azucena Herrera-González, Luis J. Figueroa-Yáñez, Javier Arrizon

**Affiliations:** 1 Department of Food Science and Technology, University of California, Davis, CA 95616, USA; kadamianmedina@ucdavis.edu; 2 Department of Chemical Engineering, Centro Universitario de Ciencias Exactas e Ingenierías, Universidad de Guadalajara, Guadalajara 44430, Mexico; mariaa.herrera@academicos.udg.mx; 3 Industrial Biotechnology Division, Unidad Zapopan, Centro de Investigación y Asistencia en Tecnología y Diseño del Estado de Jalisco, Guadalajara 45019, Mexico; lfigueroa@ciatej.mx

**Keywords:** phenolic fructosides, polyphenols, type 2 diabetes mellitus, ADMET, molecular docking

## Abstract

Enzymatic fructosylation has emerged as a strategy to enhance the hydrophilicity of polyphenols by introducing sugar moieties, leading to the development of phenolic glycosides, which exhibit improved solubility, stability, and biological activities compared to their non-glycosylated forms. This study provides a detailed analysis of the interactions between five phenolic fructosides (4MFPh, MFF, DFPh, MFPh, and MFPu) and twelve proteins (11β-HS1, CRP, DPPIV, IRS, PPAR-γ, GK, AMPK, IR, GFAT, IL-1ß, IL-6, and TNF-α) associated with the pathogenesis of T2DM. The strongest interactions were observed for phlorizin fructosides (DFPh) with IR (−16.8 kcal/mol) and GFAT (−16.9 kcal/mol). MFPh with 11β-HS1 (−13.99 kcal/mol) and GFAT (−12.55 kcal/mol). 4MFPh with GFAT (−11.79 kcal/mol) and IR (−12.11 kcal/mol). MFF with AMPK (−9.10 kcal/mol) and PPAR- γ (−9.71 kcal/mol), followed by puerarin and ferulic acid monofructosides. The fructoside group showed lower free energy binding values than the controls, metformin and sitagliptin. Hydrogen bonding (HB) was identified as the primary interaction mechanism, with specific polar amino acids such as serin, glutamine, glutamic acid, threonine, aspartic acid, and lysine identified as key contributors. ADMET results indicated favorable absorption and distribution characteristics of the fructosides. These findings provide valuable information for further exploration of phenolic fructosides as potential therapeutic agents for T2DM.

## 1. Introduction

The search for novel antidiabetic agents has become crucial in addressing the growing prevalence of diabetes worldwide. The plantae kingdom offers a diverse array of bioactive molecules, including polyphenols and phenolic compounds, which have demonstrated potential therapeutic effects [1]. Polyphenols are known for their antioxidant properties, attributed to the presence of hydroxyl and other chemical groups on their aromatic rings, allowing them to scavenge free radicals and mitigate cellular oxidative stress. For example, puerarin (daidzein-8-C-glucoside), a C-glucoside of the isoflavone daidzein extracted from the Puerarin genus, has shown anti-obesity and hypoglycemic properties, with therapeutic effects against various cardiovascular and cerebrovascular diseases [2,3]. Additionally, it has demonstrated potential applications in the treatment of diabetic retinopathy. Phlorizin (phloretin-2′-β-D-glucopyranoside), a dihydrochalcone found in apples, has been associated with diverse biological activities, including antidiabetic, anti-inflammatory, and neuroprotective effects [4]. Its mechanism of action involves the inhibition of sodium-dependent glucose transporters (SGLTs), making it a promising SGLT2 inhibitor for type 2 diabetes mellitus (T2DM) [5,6]. Ferulic acid (4-hydroxy-3-methoxycinnamic acid), an abundant hydroxycinnamic acid in various plant sources, is considered a potent antioxidant with anti-inflammatory properties [7,8].

However, the hydrophobic nature of these polyphenols poses challenges in terms of solubility and bioavailability, limiting their therapeutic applications. To overcome this limitation, enzymatic glycosylation has emerged as a strategy to enhance the hydrophilicity of polyphenols by introducing sugar moieties. This enzymatic transformation results in the formation of phenolic glycosides, which exhibit improved solubility, stability, and potentially altered biological activities compared to their non-glycosylated counterparts [9]. In recent studies, we have successfully implemented an enzymatic fructosylation process using a levansucrase (E.C. 2.4.1.10) enzyme derived from *Gluconacetobacter diazatrophicus (Gd_LsdA).* This approach has been applied to phenolic compounds such as puerarin, phlorizin, and ferulic acid, resulting in the generation of their respective phenolic fructosides. The fructosylation process not only enhanced the solubility and hydrophilicity of these compounds but also retained their antioxidant activity [10,11] to a considerable extent, with the exception of ferulic acid [12].

While the fructosylation of puerarin, phlorizin, and ferulic acid has shown promising results in terms of physicochemical properties, little is known about the specific biological effects of the resulting phenolic fructosides. In particular, their potential antidiabetic activities have not been investigated. Given the urgent public health concern of diabetes as well as the association between oxidative stress and inflammation-related diseases, it is crucial to explore the antidiabetic effects and anti-inflammatory properties of these phenolic fructosides.

Molecular docking is an in silico method for recognizing the correct binding position of a protein–ligand complex and evaluating its strength using various calculation criteria to select the best position generated by each molecule in an order of range [13]. The mean square deviation, or RMSD of atomic coordinates after the rigid body optimal superposition of two structures is undoubtedly the most popular choice for quantifying and/or validating differences in macromolecular structures and dynamics [14,15]. These tools have been used for the prediction of biological properties; for example, Chigurupati et al. (2022) determined in vitro the antidiabetic effect of a *Moringa oleifera* extract from leaves, as well as a docking analysis of their phenolic compounds. They found that this plant is a natural source of bioactive compounds against diabetes [16].

Moreover, understanding the absorption, distribution, metabolism, excretion, and toxicity (ADMET) properties of these compounds is vital for assessing their potential as drug candidates. Computational simulation and modeling, provide valuable tools for predicting the molecular interactions of these phenolic fructosides with key biological processes, such as absorption, distribution, metabolism, and excretion. Therefore, in this study, we aim to employ an in silico approach to investigate the antidiabetic effects and ADMET properties of phenolic fructosides derived from puerarin, phlorizin, and ferulic acid. By utilizing advanced computational tools, we can gain insights into the molecular interactions and potential therapeutic benefits of these compounds, thus facilitating the development of novel antidiabetic agents with improved efficacy and bioavailability. Overall, the aim of this study is to explore and elucidate the biological activities and pharmaceutical potential of phenolic fructosides as potential therapeutic alternatives for the management of T2DM and related complications.

## 2. Results

### 2.1. Synthesis and Physicochemical Properties of Phenolic Fructosides

**Figure 1:**
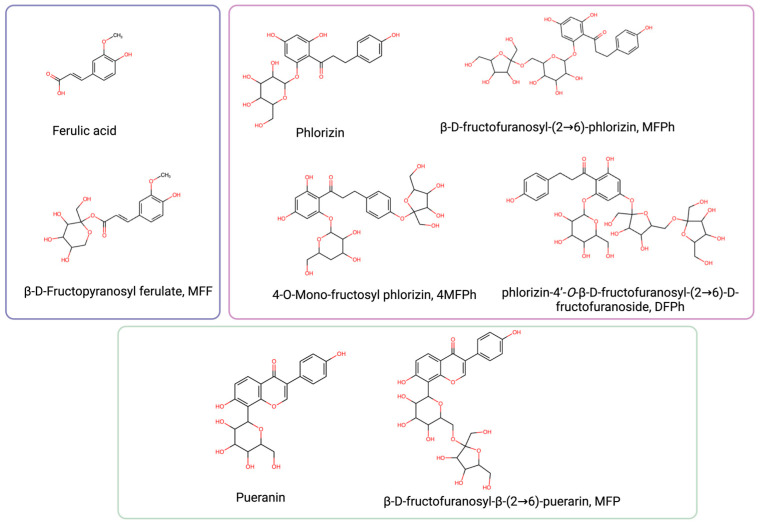
Chemical structure of ferulic acid, pueranin, phlorizin, and their fructosides (created with https://www.biorender.com accessed on 20 January 2024).

The molecular structures of ferulic acid, phlorizin, puerarin, and their respective fructosides are shown in Figure 1. As it can be seen, fructose is linked to different hydroxyls in the aglycone of ferulic acid (MFF), in the aglycone of phlorizin (4MFPh and DFPh), in glucose linked to phlorizin (MFPh), or in glucose linked to puerarin (MFP).

The reported properties of these molecules are presented in Table 1. All the fructosides increased the solubility in comparison with their respective precursors, and most of them retained the antioxidant capacity.

### 2.2. In Silico Analysis of Phenolic Fructosides

The proteins selected for docking and ADMET analyses are presented in Table 2 with their respective abbreviation, PDB code and function.

Twelve proteins (11β-HS1, CRP, DPPIV, IRS, PPAR-γ, GK, AMPK, IR, GFAT, IL-1ß, IL-6, and TNF-α) identified from the literature as playing an important role in the pathogenesis of T2DM were used to evaluate their interaction with five phenolic fructosides: 4-O-Mono-fructosyl phlorizin (4MFPh); β-D-Fructopyranosyl-β-(2→6)-ferulate(MFF); Phlorizin-4′-O-β-D-fructofuranosyl-(2→6)-D-fructofuranoside (DFPh); β-D-fructofuranosyl-β-(2→6)-phlorizin (MFPh); and β-D-fructofuranosyl-β-(2→6)-puerarin (MFPu). There are three polyphenols, ferulic acid, puerarin, and phlorizin, and two commercially available drugs, sitagliptin and metformin. In total, 120 in silico docking analyses were performed. The free energy of the binding of phenolic fructosides resulted in predictable favorable interactions with T2DM target proteins. The strongest interactions were observed for phlorizin fructosides, followed by puerarin and ferulic acid monofructosides. In the case of phlorizin fructosides, DFPh showed the highest values on proteins such as 11β-HS1 (−13.98 kcal/mol), DPPIV (−15.48 kcal/mol), IR (−16.8 kcal/mol), and GFAT (−16.8 kcal/mol). MFPh showed higher interaction values with 11β-HS1 (−13.99 kcal/mol), DPPIV (−10.77 kcal/mol), IRS (−10.49 kcal/mol), IR (−10.92 kcal/mol), and GFAT (−12.55 kcal/mol). The free energy of binding values observed between the proteins and 4MFPh were 11β-HS1 (−11.88 kcal/mol), DPPIV (−10.01 kcal/mol), IR (−11.37 kcal/mol), GFAT (−11.79 kcal/mol), and IR (−12.11 kcal/mol). The fructoside MFF showed interaction values below −10 kcal/mol with proteins such as 11β-HS1 (−8.90 kcal/mol), PPAR-γ (−9.71 kcal/mol), and AMPK (−9.10 kcal/mol) (Table 3).

Likewise, the interaction values between the non-glycosylated phenolic compounds and the proteins were lower than the glycosylated compounds; the negative values for FA averaged between −2.90 and −5.63 kcal/mol, PH −5.21 and −8.36 kcal/mol, and PU −7.50 and −10.25 kcal/mol. Regarding the interactions between the control drugs and the proteins, all the fructosides showed higher free energy binding values than sitagliptin with PPAR-γ, GFAT, 11β-HS1, and AMP K and metformin with PPAR-γ, 6K, IRS, and DPPI IV. The lowest free energy of binding values was observed for MFF when linked to PPAR-γ, AMP K, 11β-HS1, and GFAT; these values were even lower than the interactions of those proteins with sitagliptin and higher than metformin (Table 3).

The top five best molecular interactions of each compound were chosen based on their inhibitory potential, free energy binding, inhibition constant, and the type of interaction and interacting amino acid residues. Hydrogen binding (HB) was responsible for most of the highest free energy interactions, followed by polar interactions (P), and they varied in function of the type of protein and fructosides. The proteins 11β-HS1 and PPAR-γ AMPK interacted with HB; other proteins such as GFAT and IL-6 interacted through HB and P interactions, while DPP IV and IR exhibited principally P interactions. The highest free energies corresponded to phlorizin fructosides (MFPh, 4MFPh, and DFPh) with all the proteins by both HB and P interactions. The second-highest interactions corresponded to MFP with proteins 11β-HS1, GFAT, and IL-6, linked by HB and P interactions. Finally, MFF resulted in BH interactions on PPAPγ and AMPK proteins (Table 4).

Regarding the distance of the amino acids involved in the protein–fructosides interactions, the results varied depending on the type of fructoside. Serin was the closest amino acid for DFPh in 11β-HS1 (SER 142) and in GFAT (SER 401) proteins by HB interaction; the same amino acid was the closest for DPP IV protein (SER 86) by P interaction, while glutamine and glutamic acid were the closest amino acids for phlorizin fructosides in IL-6 (GLN 175) and IR (GLU 22) by HB and polar interactions, respectively. In the case of MFP, serine, threonine, and aspartic acid were the closest amino acids for 11β-HS1 (SER 12), GFAT (THR 425), and IL-6 (ASP 34), with HB interactions for the 11β-HS1 and GFAT proteins and P interactions for the IL-6 protein. Finally, the closest amino acids for MFF in the PPAPγ and AMPK proteins were glutamine (GLU 343) and lysine (LYS 141), respectively, both of them through HB interactions (Table 4).

**Figure 2:**
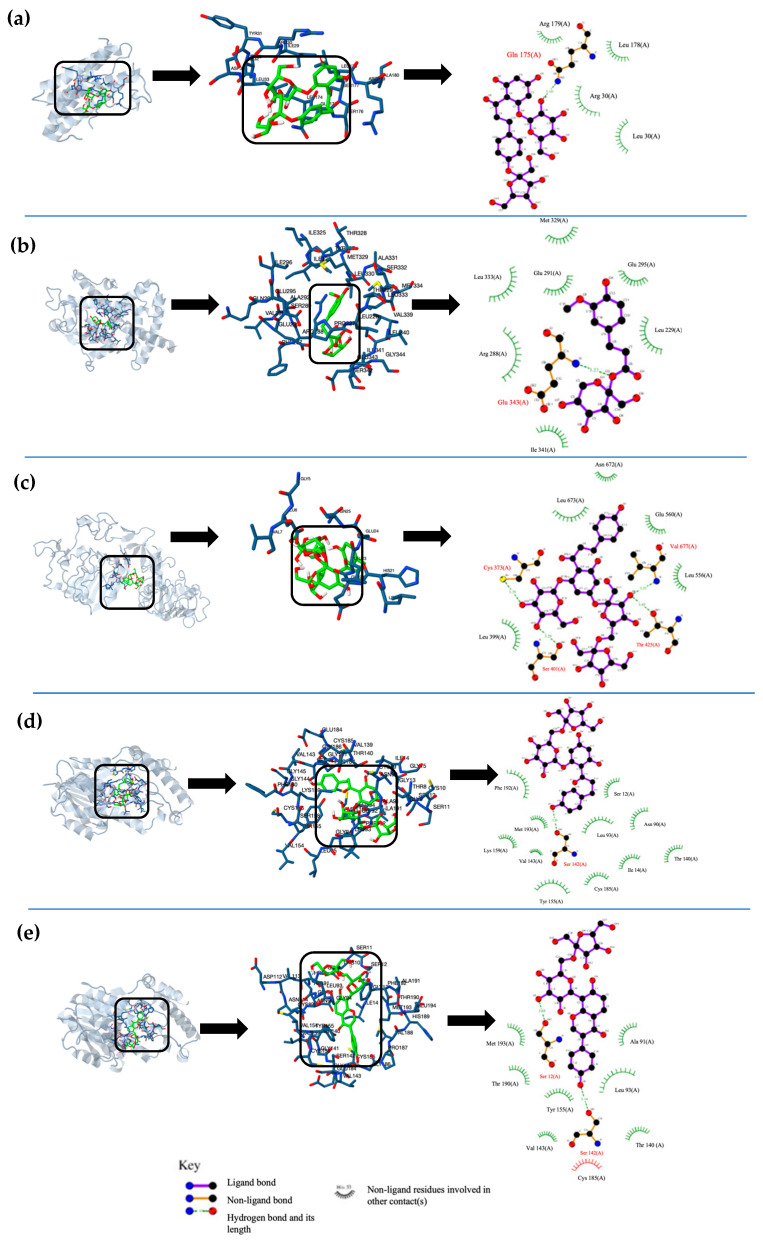
Graphic representation of the top 5 best molecular docking interactions between ligands and T2DM target proteins. (**a**) best interaction poses of 4-O -Mono-fructosyl phlorizin with IL-6, (**b**) β-D-Fructopyranosyl-β-(2→6) ferulate with PPAR-γ, (**c**) Phlorizin-4-O-β-D-fructofuranosyl-(2→6)-D-fructofuranoside with GFAT, (**d**) β-D-Fructopyranosyl-β-(2→6) phlorizin with 11β-HS1, and (**e**) β-D-Fructopyranosyl-β-(2→6) puerarin with 11β-HS1.

The interactions for each phenolic fructoside on T2DM target proteins are represented in Figure 2. In these interactions, the type of the closest amino acid, the type of molecular interaction, and the molecular site of ligands varied in the function of the fructoside. In the docking interaction of 4MFPh with IL-6, GLN interacted with the hydroxyl group of glucose by a hydrogen bond (Figure 2a). In the interaction between MFF and PPAR-γ, GLU interacted with the oxygen of the linked fructose of MFF by a hydrogen bond (Figure 2b). In the case of DFPh and IR, a GLU interacted with the linked glucose of phlorizin by polar forces (Figure 2c). For the interactions of MFPh and MFP with 11β-HS1, SER interacted by hydrogen bonds with a hydroxyl group of phlorizin aglycone and a puerarin aglycone, respectively (Figure 2d,e).

### 2.3. ADMET Properties of Phenolic Fructosides

We evaluated how the phenolic fructoside compounds behave in terms of absorption, distribution, metabolism, excretion, and toxicity (ADMET). Regarding absorption, C2P and HIA values were predicted. Phenolic fructosides like MFPh, MFP, and MFF showed lower C2P values (−6.492, −6.350 to −5.907, respectively), and all of the phenolic fructosides showed high HIA values between 0.83 and 1. Regarding distribution, the parameter PPB was measured. Compounds like 4MFPh, MFF, and MFP resulted in higher PPB percentages: 61.59%, 72.93, and 78.85%, respectively. Non-fructoside compounds like PU and PH also exhibited relatively high PPB percentages (0.93 and 0.83, respectively). The isoform CYP1A2 is a gold standard for evaluating drug metabolism and stands in a pivotal position within the cytochrome P450 family, primarily expressed in the hepatic region. CYP1A2 values for all the compounds evaluated were between 0.002 and 0.547. In terms of excretion, clearance and half-life values were measured. For both parameters, all of the phenolic fructosides showed negative results, denoted as “R”. Regarding toxicity, AMES and H-HT parameters were predicted. Almost all phenolic fructosides demonstrated an absence of AMES, affirming their low likelihood of causing genetic mutations. For H-HT, all compounds tested positive, within a range of 0.08 to 0.345, indicating a low probability of hepatotoxic effects. Exceptionally, the compound MFPh exhibited a higher value of 0.823 compared to other measured compounds, suggesting a potentially elevated hepatotoxicity probability (Table 5).

## 3. Discussion

This is the first study to investigate the potential anti-diabetic biological properties of phenolic fructosides obtained from puerarin, phlorizin, and ferulic acid in comparison with two common anti-diabetic pharmaceuticals, metformin and stagliptin. Docking analyses were performed on twelve selected proteins involved in diabetes development according to the literature, and their respective ADMET parameters were determined. Our results showed that these predictive properties varied in function of the molecular structure of fructosides in all cases. Regarding phenolic compounds, studies have demonstrated the antidiabetic potential of glycosylated phenolics in silico [16,20]. For example, Damian-Medina et al. evaluated the in silico interactions between cyanidin 3-glucoside and delphinidin 3-glucoside extracted from blue corn and black beans, respectively; the compounds were linked to key proteins in type 2 diabetes, and the strongest interactions of these two glycosylated phenolics showed free binding energies from −7.3 to −6.10 [16]. Other non-glycosylated phenolic compounds, such as phenothiazine derivatives of α-amylase, α-glucosidase, and aldose reductase, were evaluated in silico with these proteins and showed a free energy of binding between −11.3 and −5.1 kcal/mol [20]. Other compounds, such as Indole-3-heterocyclic derivatives, were evaluated on α-amylase and resulted in free energies of binding ranging from + 4.14 to −7.6 kcal/mol [21]. Finally, cannabinol, a non-glycosylated phenolic, was evaluated on the EGFR, SRC, ESR1, and HSP90AA1 diabetic proteins and showed free binding energy from −7.44 to −6.38 kcal/mol [22]. Our results showed lower free energy binding interactions of phenolic fructosides with T2DM proteins, and the strongest interactions observed were characterized by hydrogen bonds, specifically with polar amino acids (Table 4). The inhibitory constant of a drug or a compound becomes important to predict clinically relevant drug interactions by causing the inhibition of cytochrome P450 (CYP450) with small amounts of a medication or compound [23]. As for the inhibition constant (Ki), the interactions between 4MFPh with IR (9.87) and IL-6 (1.33), DFPh with DPPIV (4.52), and MFP with 11β-HS1 (1.55) showed inhibitory potential by scoring the lowest Ki values. Prior research has shown that polyphenols such as epigallocatechin gallate (EGCG) have the potential to significantly inhibit various cytochrome P450 isoforms in human liver microsomes, including CYP2C9 (7.60 µM) and CYP1A2 (8.93 µM). In addition, the calculated Ki values indicated that EGCG could potentially modulate the metabolism of drugs that are substrates for CYP3A4 [24]. Several in silico studies have demonstrated the potential inhibitory action of different antioxidants. For example, a study revealed that resveratrol has significant inhibitory activity against α-amylase, an enzyme crucial in carbohydrate digestion, with better Ki values than the reference drug acarbose [25]. Additionally, another study showed that the hydroxyl at C7 interfered with the tyrosinase activity of the flavonoid quercetin and increased the anti-tyrosinase activity compared to kojic acid, used as a control inhibitor [26].

Regarding the amino acids involved in the protein interactions with different phenolics, Damian-Medina et al. (2020) [16] demonstrated the strongest in silico interactions with polar and non-polar amino acids by hydrogen bonds. For example, cyanidin 3-glucoside interacted with SER, GLY, and TYR for the 11β-HS1 protein, GLN, ASP, ASN, and GLY for the GFAT protein, and VAL, GLU, and PRO for the PPARG protein, while delphinidin 3-glucoside interacted with LYS, GLY, and ASN for the 11β-HS1 protein, SER, ASP, ASN, and GLU for the GFAT protein, ASP, ARG, LYS, and TYR for the PTP protein, and VAL and PRO for the RTK protein. In the present study, the phenolic fructosides interacted principally with polar amino acids by hydrogen bonds, possibly because they are highly hydroxylated by fructosylation. Another possible explanation is that serine emerged as the amino acid closest to proteins in most interactions. This has been observed in other studies, where the strongest interactions were found for TRP, ASP, and ASP amino acids with α-amylase and for ARG, ASP, ASP, and SER amino acids with α-glucosidase [20]. However, other studies using other phenolic compounds such as phenothiazine, indole 3-heterocyclic, and cannabinol demonstrated higher interactions with non-polar amino acids [20,21,22]. According to Zhou et al., the position of hydroxyl groups in flavonoids determines not only the biological properties, but it also influences the interaction with proteins, in particular the hydroxyl groups of the ring B [27]. Regarding the amino acids involved in the interaction with phenolics, it depends on the molecular structure of these compounds; in general, phenolics interact with aromatic amino acids such as phenylalanine, while alkyl phenols interact with hydrophobic amino acids with long chains like proline, leucine, and phenylalanine [27].

The molecular docking results indicated that the best interaction of phenolic fructosides was with proteins 11β-HS1, GFAT, IL-6, DPPIV, PPAR-γ, AMPK, and IR. These proteins are part of a complex network of molecular events that contribute to the pathophysiology of T2DM. The enzyme 11β-HSD1 plays a key role in the regulation of glucocorticoid levels in adipose tissue and the liver and converts inactive cortisone to active cortisol. Cortisol promotes lipolysis, which results in the release of free fatty acids (FFAs). Elevated FFAs contribute to insulin resistance caused by impaired insulin signaling and increased inflammation; additionally, cortisol inhibits glucose uptake in peripheral tissues and further contributes to insulin resistance [27]. GFAT is an enzyme involved in the hexosamine biosynthetic pathway that catalyzes the conversion of fructose-6-phosphate to glucosamine-6-phosphate in the hexosamine biosynthetic pathway (HBP). Increased HBP activity leads to the production of uridine diphosphate N-acetylglucosamine (UDP-GlcNAc), elevated UDP-GlcNAc levels contribute to insulin resistance as a result of the modification of insulin signaling proteins through O-GlcNAcylation, disrupting normal insulin signaling [28]. Inflammation, often marked by elevated IL-6 levels, contributes to insulin resistance. IL-6 activates the Janus kinase-signal transducer and activator of transcription (JAK-STAT) pathway and suppressor of cytokine signaling (SOCS) proteins that produce an alteration in insulin receptor signaling. IL-6 also promotes the release of other proinflammatory cytokines; this produces a chronic inflammatory environment detrimental to insulin sensitivity [29]. DPPIV is an enzyme involved in the regulation of incretin hormones, which play a role in glucose homeostasis. DPPIV cleaves incretin hormones, such as GLP-1, and rapidly degrades them. The inhibition of DPPIV increases the half-life of GLP-1 and allows it to exert its effects for a more extended period. GLP-1 enhances insulin secretion, inhibits glucagon release, delays gastric emptying, and promotes satiety. The prolonged actions of GLP-1 and DPPIV inhibitors improve glucose homeostasis [30]. PPAR-γ is a nuclear receptor involved in the regulation of glucose and lipid metabolism that forms a heterodimer with the retinoid X receptor (RXR). This complex binds to PPAR-γ response elements (PPREs) in target genes and regulates adipocyte differentiation and lipid metabolism. Thiazolidinediones, PPAR-γ agonists, enhance PPAR-γ activity and promote insulin sensitivity caused by the improvement of adipose tissue function, reduced inflammation, and enhanced glucose uptake in peripheral tissues [31]. AMPK is a cellular energy sensor that helps regulate glucose and lipid metabolism and is activated when cellular AMP levels increase relative to ATP. Activated AMPK phosphorylates target proteins involved in energy metabolism. In T2DM, AMPK activation enhances insulin sensitivity, produces glucose uptake in skeletal muscles, inhibits hepatic gluconeogenesis, and regulates lipid metabolism. Additionally, AMPK has anti-inflammatory effects through the suppression of proinflammatory signaling pathways [32]. Finally, the insulin receptor (IR) is a key player in glucose homeostasis. Insulin resistance occurs when there is a reduced response to insulin signaling. This can result from serine phosphorylation of insulin receptor substrates (IRS), inhibition of the PI3K/Akt pathway, and activation of negative regulators like JNK and IKK. Dysregulation in the insulin signaling cascade leads to impaired glucose uptake and contributes to T2DM [33].

The processes by which substances are absorbed, metabolized, and excreted from the body are considered crucial. The study of these aspects is complicated due to the molecular complexity of foods rich in polyphenols, along with factors such as the degree of polymerization and conjugation with other compounds and phenols. Polyphenols in food are typically present in the form of esters, glycosides, or polymers, and cannot be absorbed in these forms. Once absorbed, polyphenols are identified by the body as xenobiotics, resulting in their relatively low bioavailability compared to micro- and macronutrients. A comprehensive understanding of these complexities is considered essential to understanding the complexity of polyphenol bioavailability [34]. The ADMET profile is crucial in drug development and various fields of scientific research. These studies provide valuable insights into how a substance, typically a drug or a chemical compound, behaves within a living organism.

In terms of the ADMET profile, phenolic fructosides such as MFPh, MFP, and MFF exhibited lower Caco-2 Permeability (C2P) values compared to control phenolic compounds and drugs. This finding suggests that fructosylation may enhance permeability and indicate a higher likelihood of absorption [35]. Fang et al. evaluated the transepithelial permeability of 30 flavonoids using a Caco-2 cell monolayer model and employed a QSPR model to predict the intestinal absorption of flavonoids based on their structural characteristics. Their findings revealed that the presence of a 3-OH or glycosidic group was associated with decreased flavonoid absorption, and equal substitutions on the A and B ring were found to be favorable for absorption [36]. In our study, all phenolic fructosides demonstrated elevated Human Intestinal Absorption (HIA) values, implying efficient absorption through the intestinal wall and facilitating entry into the systemic circulation following oral administration. The pharmacokinetics study of sixteen antidiabetic flavonoids revealed consistent findings for flavonoids such as chrysin, wogonin, baicalein, genistein, and apigenin. These compounds exhibited higher HIA levels, indicating improved absorption [37].

Notably, compounds like 4MFPh, MFF, and MFP exhibited higher plasma protein binding (PPB) percentages, indicating an increased propensity to bind to proteins in the bloodstream. This binding could influence the distribution, availability, and duration of action in the body. Conversely, some compounds like PU and PH displayed relatively high PPB percentages, potentially impacting their distribution and availability, which may be advantageous for target sites requiring elevated plasma levels. Recent research has extensively examined the relationship between PPB and phenolics, with a predominant emphasis on elucidating structure-affinity connections [38].

The isoform CYP1A2, a pivotal member of the cytochrome P450 family primarily expressed in the hepatic region, plays a crucial role in the biotransformation of various pharmacologically significant compounds. CYP1A2 values for all evaluated compounds ranged from 0.002 to 0.547, indicating potential interactions with drugs metabolized by CYP1A2. Polyphenols and flavonoids are recognized for their ability to inhibit CYP1A2. The bioflavonoid pinocembrin, frequently used in complementary/alternative medicine, demonstrated robust inhibitory activity against CYP1A2 [39]. Other compounds from edible plants such as lemongrass (*Cymbopogon citratus* (DC.) Stapf), chicory (*Cichorium intybus* L.), moringa (*Moringa oleifera* Lam.), and ryegrass (*Lolium perenne* L.) have been tested as inhibitors of CYP1A2. However, the predicted results show that none of the bioactive substances inhibited this enzyme [40].

Concerning clearance values, all phenolic fructosides exhibited negative results when compared to established diabetes treatments like metformin and sitagliptin. Slower clearance could extend compound exposure, potentially leading to prolonged effects. The longer half-life observed for all studied compounds implies their persistence in the body before elimination, impacting dosing frequency and treatment regimens. Finally, almost all phenolic fructosides demonstrated an absence of mutagenicity, affirming their low likelihood of causing genetic mutations, a crucial aspect for safety considerations.

## 4. Materials and Methods

### 4.1. Synthesis and Physicochemical Properties of Phenolic Fructosides

Mono-fructosyl puerarin (β-D-fructofuranosyl-β-(2→6)-puerarin, MFP), Mono-fructosyl phlorizin (β-D-fructofuranosyl-(2→6)-phlorizin, MFPh), 4-O-Mono-fructosyl phlorizin (4MFPh), and phlorizin difructoside (phlorizin-4′-*O*-β-D-fructofuranosyl-(2→6)-D-fructofuranoside, DFPh) were synthesized, purified, and structurally characterized according to the methodology reported by Nuñez-López et al. 2019 and Herrera-González et al. 2021 [10,11]. β-D-Fructopyranosyl ferulate (MFF) was synthesized using the levansucrase *Gd*_LsdA from *Gluconacetobacter diazotrophicus*. The reaction was carried out using 1.5 M sucrose, 25 mM ferulic acid, and 5 U mL^−1^ of enzyme in 50 mM phosphate buffer at pH 5.8 at 42 °C for 24 h. The percentage of conversion was 36% conversion. The reaction mixture was subjected to column chromatography using a Sigma-Aldrich C18 resin column (30 × 2 cm). The column was washed with pure water to remove sugars. Ferulic fructoside was eluted using an Acetonitrile/H_2_O solution (20:80 *v*/*v*). The purification process was monitored by thin layer chromatography (TLC) on silica gel 60 RP-18 F254 plates using a water/acetonitrile mixture (80:20) as the mobile phase. The TLC plates were exposed to UV light (254 nm) to visualize ferulic fructoside. Finally, the ferulic fructoside was characterized by ^1^H and ^13^C NMR.

### 4.2. Molecular Docking Analysis

#### 4.2.1. T2DM Key Protein Selection and Preparation

The key target proteins (11β-HS1, CRP, DPPIV, IRS, PPAR-γ, GK, AMPK, IR, GFAT, IL-1ß, IL-6, and TNF-α) implicated in the pathogenesis of T2DM and their respective biological functions were identified in reviews and original research articles related to T2DM. The criteria to select the key proteins were their (a) biological relevance, by choosing proteins that are directly involved in the biological processes or pathways relevant to the research question. In our case, we included proteins associated with insulin signaling, glucose metabolism, or inflammation-related pathways; (b) experimental evidence, selecting proteins with well-established experimental evidence of their involvement in the pathogenesis of T2DM; (c) protein availability and reliability, ensuring that the three-dimensional structures of the selected proteins were available in the Protein Data Bank (PDB) database (https://www.rcsb.org accessed on 10 August 2023); (d) protein function, looking for proteins with known functions that align with the research objectives; (e) protein druggability, by considering protein properties such as the presence of active sites, binding pockets, or known ligand interactions; (f) validation studies, looking for proteins that have been previously targeted or studied in drug discovery or molecular docking studies.

Although there are too many important proteins involved in T2DM, we selected a total of twelve proteins. The three-dimensional structures of these proteins were downloaded in .pdb format from the Protein Data Bank (PDB) database (https://www.rcsb.org/pdb/home/home.do accessed on 15 August 2023). The ID’s of the selected proteins are listed in Table 2. The proteins were prepared as follows; essential hydrogen atoms, Kollman united atom type charges, and solvation parameters were added with the aid of the AutoDock Vina 1.2.0 tool. Affinity (grid) maps of 20 × 20 × 20 Å grid points, and 0.375 Å spacing were generated using Autogrid. AutoDock Vina parameter set- and distance-dependent dielectric functions were used in the calculation of the van der Waals and the electrostatic terms, respectively [41].

#### 4.2.2. Molecular Docking Simulations

To investigate the potential interactions between the phenolic fructoside ligands and key proteins involved in T2DM pathogenesis, molecular docking studies were conducted. We used the .pdb files of 4-O -Mono-fructosyl phlorizin, β-D-Fructopyranosyl ferulate, Di-Fructoside, Mono-fructosyl phlorizin, and Mono-fructosyl puerarin as our experimental ligands. The chemical structures of ferullic acid, phlorizin, puerarin, metformin and sitagliptin were downloaded in .pdb format from PubChem (https://pubchem.ncbi.nlm.nih.gov accessed on 15 August 2023) and used as our control ligands. The MMFF94 force field [42] was used for the energy minimization of ligand molecules using DockingServer. Gasteiger partial charges were added to the ligand atoms. Non-polar hydrogen atoms were merged, and rotatable bonds were defined.

For docking calculations, two software tools were employed: DockingServer (https://www.dockingserver.com/ accessed on 10 August 2023) [43] and BIOVIA Discovery Studio 2017 (http://www.3dsbiovia.com/ accessed on 20 August 2023). DockingServer provided a platform for conducting docking simulations, while BIOVIA Discovery Studio 2017 was utilized for visualization and design purposes to enhance the comprehensiveness of the interactions. Docking simulations were performed using the Lamarckian genetic algorithm (LGA) and the Solis and Wets local search method [44]. The initial position, orientation, and torsions of the ligand molecules were set randomly. Each docking experiment was derived from 100 different runs that were set to terminate after a maximum of 2,500,000 energy evaluations. The population size was set at 150. During the search, a translational step of 0.2 Å, quaternion, and torsion steps of 5 were applied.

### 4.3. Absorption, Distribution, Metabolism, Excretion, and Toxicity (ADMET) Interactions

The evaluation of bioavailability, pharmacokinetics (PK), pharmacodynamics (PD), and toxicity is crucial for determining the potential success or failure of drug candidates. In our study, we aimed to assess the ADMET (absorption, distribution, metabolism, excretion, and toxicity) properties of each phenolic fructoside using the ADMETlab 2.0 module, an open-source resource available at https://admetmesh.scbdd.com (accessed on 28 August 2023). To determine the ADMET properties, all ligands, including experimental compounds and controls, were converted into SMILES format. The SMILES information was then uploaded to the ADMETlab 2.0 portal for analysis. A comprehensive set of in silico tools, including (a) Drug-likeness Evaluation, (b) ADMET Prediction, (c) Systemic Evaluation, (d) ApplicationDomain and (e) Aggregator prediction, were measured. We evaluated drug-likeness and the prediction of 31 ADMET endpoints, encompassing physicochemical properties, absorption, distribution, metabolism, elimination, and toxicity. Finally, a comprehensive file was generated, containing the ADMET properties of each ligand, as presented in Table 4.

## 5. Conclusions

This study is the first to explore the anti-diabetic properties of phenolic fructosides derived from puerarin, phlorizin, and ferulic acid, in comparison to the conventional pharmaceuticals metformin and sitagliptin. Our in silico docking analyses and ADMET profiling revealed that phenolic fructosides exhibit varying degrees of interaction with diabetes-related proteins, primarily forming strong hydrogen bonds with polar amino acids and lower free energy binding interactions. These interactions suggest potential therapeutic benefits for diabetic patients. The ADMET profiles of these fructosides, which showed efficient intestinal absorption and favorable plasma protein binding, highlight their potential as viable anti-diabetic agents. In addition, the absence of mutagenicity in these compounds supports their safety profile, encouraging further research into their clinical application for T2DM management.

## Figures and Tables

**Table 1:** Physicochemical properties of fructosides and precursors.

Compound	Solubility g L^−1^	DPPH % Free Radical Scavenging Activity	ABTS % Free Radical Scavenging Activity	NO % Free Radical Scavenging Activity
Ferulic acid (FA)	0.354 ± 0.02	70.04 ± 2.3	90.69 ± 0.14	22.21 ± 1.39
β-D-Fructopyranosyl-β-(2→6)- ferulate (MFF)	8.69 ± 0.45	17.16 ± 0.81	90.95 ± 0.18	3.27 ± 1.02
Puerarin (PU)	0.7 ± 0.09	33.3%	NR	NR
β-D-Fructofuranosyl-β-(2→6)-puerarin (MFP)	16.2 ± 1.7	26.2 ± 1.3%	NR	NR
Phlorizin (PH)	1.93 ± 0.03	62.13 ± 1.8%	NR	NR
D-β-D-Fructofuranosyl-(2→6)-phlorizin (MFPh)	30.57 ± 0.1	44.56 ± 8.4%	NR	NR
4-O-Mono-fructosyl phlorizin (4MFPh)	NR	NR	NR	NR
Phlorizin-4′-*O*-β-D-fructofuranosyl-(2→6)-D-fructofuranoside (DFPh)	NR	NR	NR	NR

NR: No reported.

**Table 2:** Biological function of selected target proteins involved in type 2 diabetes mellitus signaling pathways.

Protein	Abbreviation	PDB Code	Function
Glucokinase	GK	1V4S	Catalyzes the transfer of phosphate from ATP to glucose to generate glucose 6-phosphate [17].
AMP-activated protein kinase	AMPK	2H6D	Involved in the stimulation of glucose transport and fatty acid oxidation [17].
11 β-hydroxysteroid dehydrogenase 1	11β-HS1	1BHS	Produces insulin resistance throuh the conversion of cortisone to cortisol [17,18].
Insulin receptor substrate	IRS	1K3A	Impairment of IRS-2 signaling in the β-cell produces β-cell loss in T2DM [18].
Interleukin 1 beta	IL-1ß	9ILB	Contributes to inflammation of beta cells in pancreas [19].
Dipeptidyl peptidase IV	DPPIV	1J2E	Inhibits the action of GIP and GLP-1, increasing glucose levels [17].
C-reactive protein	CRP	1GNH	Involved in chronic inflammation in adipose tissue and leads to insulin resistance [19].
Glutamine fructose-6-phosphate amidotransferase	GFAT	2ZJ3	Increased the flux of glucose through the pathway where GFAT is a key catalyst that can lead to insulin resistance [17,18].

**Table 3:** Molecular docking results from the interaction between polyphenols and key proteins in T2DM pathways. The results are expressed as free energy of binding (kcal/mol).

Ligands	Proteins											
	11β-HS1	CRP	DPPIV	IRS	PPAR-γ	GK	AMPK	IR	GFAT	IL-1ß	IL-6	TNF-α
4-O-Mono-fructosyl phlorizin (4MFPh)	**−11.88**	+864.05	**−10.01**	−6.62	−5.79	+13.38	−3.49	**−11.37**	**−11.79**	+606.71	**−12.11**	+141.26
β-D-Fructopyranosyl-β-(2→6)- ferulate(MFF)	**−8.90**	+110.52	−6.45	−7.36	**−9.71**	−7.69	**−9.10**	−6.76	−8.14	+27.95	−6.95	−2.21
Phlorizin-4′-O-β-D-fructofuranosyl-(2→6)-D-fructofuranoside (DFPh)	**−13.98**	+1.64	**−15.48**	+6.54	+13.34	+107.26	+16.42	**−16.8**	**−16.9**	+950.39	+53.21	+470.95
Ferulic acid (FA)	−4.78	+4.48	−2.90	−3.60	−5.01	−4.64	−5.26	−4.24	−4.28	−4.21	−4.58	−5.63
ß-D-fructofuranosyl-ß-(2→6)-phlorizin (MFPh)	**−13.99**	+915.68	**−10.77**	**−10.49**	−5.21	+22.38	−7.66	**−10.92**	**−12.55**	+407.72	−6.80	+113.82
ß-D-fructofuranosyl-ß-(2→6)-puerarin (MFPu)	**−12.02**	+775.58	**−9.90**	−7.85	**−9.17**	+23.3	−6.47	**−10.22**	**−10.26**	+326.27	**−10.29**	+184.51
Phlorizin (PH)	−8.36	+321.76	−5.65	−6.97	−8.61	+0.73	−5.77	−5.21	−8.01	+139.38	−5.88	+8.28
Puerarin (PU)	−10.25	+498.85	−7.50	−8.24	−11.76	+7.73	−7.66	−7.57	−8.96	+213.72	−8.01	+76.95
Metformin	−3.18	−3.87	−4.84	−4.97	−6.06	−5.79	−4.00	−4.83	−4.37	−3.80	−3.43	−2.62
Sitagliptin	−8.90	+133.74	−7.47	−8.30	−12.73	−7.60	−8.58	−7.30	−10.39	+28.82	−7.28	−7.41

The values in bold represent the lowest free energy of binding (kcal/mol) between the ligands and the proteins.

**Table 4:** Summary of the physicochemical characteristics of the three top interactions of each ligand.

Ligand	Protein	Free Energy of Binding (kcal/mol)	Inhibition Constant (Ki) nM	Type of Interaction	Amino Acid Residue Interaction
4-O-Mono-fructosyl phlorizin (4MFPh)	11β-HS1	−11.88	1.96	HB	O14-SER11 [2.75]; O4-TYR155 [2.88]; H31-SER11 [2.70]; H31-SER12 [3.64]
	GFAT	−11.79	2.28	P	O2-SER401 [3.69]; H9-SER401 [3.76]; O15-SER422 [3.87]; O2-ASP427 [3.00]; H9-ASP427 [2.32]; O7-GLU560 [3.53]; H15-GLU560 [3.89]
	IL-6	−12.11	1.33	HB	O9-GLN175 [3.10]
β-D-Fructofuranosyl-β -(2→6) phlorizin (MFPh)	11β-HS1	−13.91	55.28	HB	O7-SER142 [3.26]; H20-SER142 [2.63]; H20-VAL143 [3.27]
	GFAT	-12.55	627.45	HB	O15-CYS373 [3.25]; O2-ALA674 [3.27]; H28-CYS373 [3.58]; H28-ASP427 [3.61]
	IR	−10.92	9.87	P	O11-GLU24 [3.41]; O10-GLU24 [3.63]; H21-GLU24 [3.82]; O5-ASN25 [3.32]; O9-ASN25 [3.36]; H22-ASN25 [3.86]; H10-ASN25 [3.77]
Phlorizin-4′-O-β-D-fructofuranosyl-(2→6)-D-fructofuranoside (DFPh)	DPPIV	−15.48	4.52	P	O7-ASN85 [3.56]; O15-ASN85 [3.57]; O1-SER86 [3.35]; H9-SER86 [2.62]; H10-SER87 [3.64]
	IR	−16.80	481.43	P	H44-GLU6 [2.73]; O20-GLU6 [3.02]; O2-GLU22 [2.92]; H10-GLU22 [1.94]; O3-GLU24 [3.42]
	GFAT	−16.87	431.06	HB	O18-CYS373 [3.75]; O17-SER401 [3.25]; O18-SER401 [3.23]; O6-THR425 [3.43]; O6-VAL677 [3.37]; H38-LEU399 [3.81]; H37-SER401 [3.68]; H38-SER401 [3.18]; H21-THR425 [3.46]
β-D-Fructopyranosyl-β-(2→6) ferulate (MFF)	11β-HS1	−8.9	298.32	HB	O9-TYR155 [3.00]; O8-TYR155 [2.87]; O4-LYS159 [3.47]; H13-TYR155 [3.88]; H22-TYR155 [3.36]; H21-TYR155 [3.20]
	PPAR-γ	−9.71	76.66	HB	O2-GLU343 [3.27]
	AMPK	−9.1	213.35	HB	O7-LYS141 [3.02]; O4-ASP157 [3.26]; H13-LYS45 [3.59]; H20-LYS141 [3.53]; H20-ASN144 [3.32]; H13-ASP157 [3.51]
β-D-Fructofuranosyl-β-(2→6)-puerarin (MFP)	11β-HS1	−12.02	1.55	HB	O7-SER12 [2.88]; O3-SER142 [3.14]; H16-SER12 [2.91]; H8-SER142 [3.32]; H8-VAL143 [3.73]
	GFAT	−10.26	30.14	HB	O7-THR425 [3.24]; H16-THR425 [3.51]
	IL-6	−10.29	28.47	P	O13-ARG30 [3.52]; O4-ARG30 [3.27]; H9-ARG30 [3.75]; O14-ARG30 [3.48]; H30-ARG30 [3.33]; H27-ARG30 [3.85]; O11-ASP34 [3.01]; H23-ASP34 [2.06]; O12-ASP34 [3.01]; H24-ASP34 [3.72]; O8-GLN175 [3.11]; H17-GLN175 [3.66]; O2-ARG182 [3.51]

**Table 5:** ADMET profile of polyphenols and commercially available drugs for T2DM treatment.

Ligands	Absorption				Distribution		Metabolism		Excretion				Toxicity			
	C2P	R	HIA	R	PPB (%)	R	CYP1A2 Inhibitor	R	CL	R	T ½	R	AMES	R	H-HT	R
4-O-Mono-fructosyl phlorizin (4MFPh)	−6.505	+	0.998	+	61.59	+	0.016	+	3.15	−	0.436	−	0.125	−	0.159	+
Phlorizin-4-O-β-D-fructofuranosyl-(2→6)-D-fructofuranoside (DFPh)	−6.634	+	1.0	+	37.56	+	0.002	+	1.79	−	0.362	−	0.083	−	0.125	+
*β-D-Fructopyranosyl- β-(2→6)* phlorizin (MFPh)	−6.492	+	0.997	+	57.84	+	0.013	+	4.33	−	0.54	−	0.161	−	0.823	+
β-D-Fructopyranosyl- β-(2→6) ferulate (MFF)	−5.907	+	0.935	+	72.93	+	0.023	+	5.67	−	0.855	−	0.086	−	0.133	+
β-D-Fructopyranosyl- β-(2→6) puerarin (MFP)	−6.350	+	0.989	+	78.88	+	0.009	+	2.58	−	0.352	−	0.353	−	0.084	+
Phlorizin	−6.318	+	0.953	+	65.29	+	0.104	+	8.40	−	0.706	−	0.523	−	0.057	+
Puerarin	−6.038	+	0.83	−	89.30	+	0.046	+	2.68	−	0.491	−	0.531	−	0.08	+
Ferulic acid	−4.902	−	0.03	−	89.75	+	0.059	+	7.48	−	0.926	−	0.224	−	0.345	+
Metformin	−8.670	+	1.0		55.34	+	0.365	+	1.25	+	0.856	−	0.496	+	0.245	−
Sitagliptin	0.536	+	0.989		34.56	+	0.547	+	3.25	+	0.478	-	0.148	+	0.152	+

C2P: Caco-2 permeability; HIA: Human Intestinal Absorption; PPB: plasma protein binding; CYP1A2: cytochrome P450 phase 1; CL: clearance; T ½: half-life; AMES: mutagenicity; H-HT: human hepatotoxicity; R: Result (+: Positive, −: Negative).

## Data Availability

Data are contained within the article.
